# Operation and management of a community treatment center using telemedicine for foreign patients with mild COVID-19 symptoms

**DOI:** 10.1097/MD.0000000000027948

**Published:** 2021-11-24

**Authors:** Han Bit Kim, Sangsoo Han, Heejun Shin, Young Hwan Lee, Kyung Min Lee, Jae Ryoung Kwak, Young Soon Cho, Hojung Kim, Hoon Lim, Gi Woon Kim, Eunkyung Eo, Hyun Noh

**Affiliations:** Department of Emergency Medicine, Soonchunhyang University Bucheon Hospital, Bucheon, Republic of Korea.

**Keywords:** community treatment center, COVID-19, medical resources, pandemic, telemedicine

## Abstract

South Korean studies on coronavirus disease-2019 (COVID-19) treatment have described the use of community treatment centers (CTCs), which combine elements of the home and hospital, to isolate and treat mild COVID-19 patients. While the number of South Koreans diagnosed with COVID-19 cases has varied greatly by season, the number of confirmed cases in foreign nationals has shown no seasonality, with an average of around 25 to 30 per day. For foreign patients, accommodation arrangements and travel routes may be difficult; they may also have difficulty accessing medical care, so require careful management.

We discuss our experience in operating and managing a CTC for foreign COVID-19 patients arriving in South Korea with mild symptoms. We also propose guidelines for efficient use of resources with respect to treating these patients in CTCs.

We present the clinical findings of patients treated at the CTC between 7 October and 22 November 2020, and make some recommendations. We quarantined and treated foreign patients with mild symptoms of COVID-19 at the Ansan CTC. Discharge is determined based on clinical symptoms rather than polymerase chain reaction results. Medical and administrative staff use building A, while building B is used for isolating patients. Medical rounds are in the form of twice-daily video calls. Three kinds of foods with medication are served according to the patient's country of origin.

In total, 315 patients were admitted to the Ansan CTC between 7 October and 22 November 2020; 145 of them were discharged from the CTC and 26 were transferred to other hospitals.

To utilize medical resources efficiently during the pandemic, it is desirable to reserve CTCs exclusively for foreign patients.

## 1 Introduction

Coronavirus disease-2019 (COVID-19) spread around the world in 2020 after the initial outbreak in Wuhan, China in 2019.^[1]^ Many countries are now facing a serious situation due to a lack of medical resources. In Korea, 34,201 patients had been confirmed as COVID-19-positive by 30 November 2020, following the first case of a Chinese woman who arrived from Wuhan, China, on 19 January 2020. There have been 29,651 South Korean COVID-19 patients and 4,550 from overseas.^[2]^ Currently, critically ill COVID-19 patients are hospitalized and treated in designated hospitals, while those with mild or no symptoms are treated in isolation in community treatment centers (CTCs).^[3,4]^ It is important to treat COVID-19 patients entering South Korea from other countries. Since 12 June 2020, all asymptomatic overseas patients have self-isolated and been tested for COVID-19 within 3 days of arrival in South Korea; those positive for COVID-19 have been divided into 2 groups: high-risk and mild/asymptomatic. The National Medical Center provides beds for high-risk patients. Mild and asymptomatic patients are mainly assigned to temporary patient facilities called CTCs, which combine elements of the home and hospital environment. Patients are isolated and monitored in the CTCs, which were designed by the Korean government.^[3]^ There have been several reports describing the principles and operation of CTCs in Korea.^[3–7]^

The international migrants who need to cross borders, even during the COVID-19 pandemic, are mainly students and workers. Around 1.5 million people are moving around the world.^[8]^ For foreign patients, accommodation arrangements and travel routes may be unknown; access to medical care may also be limited, so careful management is required.

There were 769 COVID-19 patients on cruise ships in Japan in February 2020. The patients were classified according to severity and assigned to hospitals. Patients with mild or no symptoms were also treated in isolation at the hospital and categorized into the high-risk group.^[9]^ However, there have been no other reports on the management of mild and asymptomatic overseas patients. China built a new quarantine hospital for such patients, while other countries implemented self-quarantine measures without hospitalization.^[10,11]^

As discussed above, although the patient-management activities of CTCs have been reported, this has not extended to those entering South Korea from abroad. In addition, there have been no reports of non-face-to-face management of COVID-19 patients in CTCs. Thus, we present recommendations for managing mild and asymptomatic overseas COVID-19 patients.

## 2 Methods

In February 2020, there was a major outbreak of COVID-19 in places of worship in Daegu, South Korea; in response, the first CTC was opened in South Korea to address the shortage of medical facilities.^[5]^ Existing accommodation facilities were used as CTCs beginning in March 2020.^[12]^ Chest X-ray and real-time polymerase chain reaction (RT-PCR) analyses were conducted, and telemedicine in the form of smartphone video calls was used to successfully minimize the spread of infection. However, the importance of managing overseas migrants has increased due to the increasing prevalence of COVID-19 in Europe, the United States, and other territories, including Asia. Overseas COVID-19 patients are classified based on symptoms (symptomatic or asymptomatic), nationality (Korean or non-Korean), stay duration (long- or short-term), and the requirement for quarantine. A diagnostic test is conducted for all non-Korean individuals arriving from overseas except flight attendants. Self-quarantine and active monitoring are performed for those who are negative for COVID-19. COVID-19-positive patients are classified as severe or mild and provided with a hospital bed.

This study is retrospective observational study and was approved by the hospital institutional trial review board (IRB File No. 2020-12-030-001).

### 2.1 Admission and discharge

**Figure 1:**
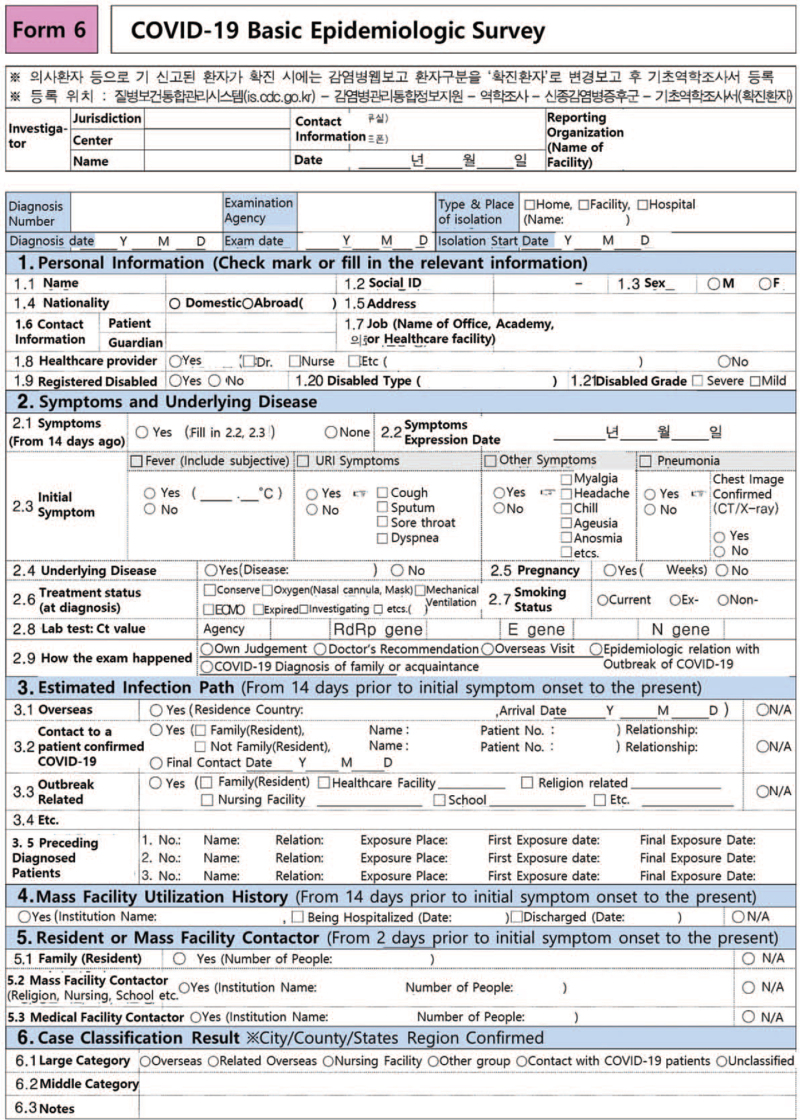
Basic epidemiologic survey, the survey to fill out before patients come to the community treatment center.

The Ansan CTC was designed for patients with mild or no symptoms according to the guidelines of the Korea Centers for Disease Control and Prevention (KCDC).^[13]^ The criteria for asymptomatic patients are as follows: alert, <50 years old, no underlying disease, nonsmoker, and body temperature <37.5°C without antipyretic drugs. The criteria for mild symptoms are as follows: alert, <50 years old, 1 or more underlying diseases, and body temperature <38°C with antipyretic drugs (Fig. 1).

The admission process is as follows. First, after being identified at a local health center, the patient is informed of the quarantine arrangements. Second, a “facility allocation request” is made after assessing disease severity. Patients are then classified and assigned to a facility. Third, the local health center transports the patient via public ambulance; the paramedics and drivers wear level D protective clothing during this process. Finally, patient care is transferred to the CTC; if the symptoms worsen, the patient is transferred to a designated hospital.

Discharge is determined based on clinical symptoms rather than PCR results. Asymptomatic patients are discharged 10 days after diagnosis if no symptoms are observed during the hospitalization period. Patients with mild symptoms can be discharged if symptoms show improvement and there has been no fever for 72 hours at 10 days after symptom onset. The criteria for discharge are those of the KCDC based on the report that viral culture remaining negative at about 8 to 10 days after the onset of COVID-19 symptoms.^[6]^

### 2.2 Facility

**Figure 2:**
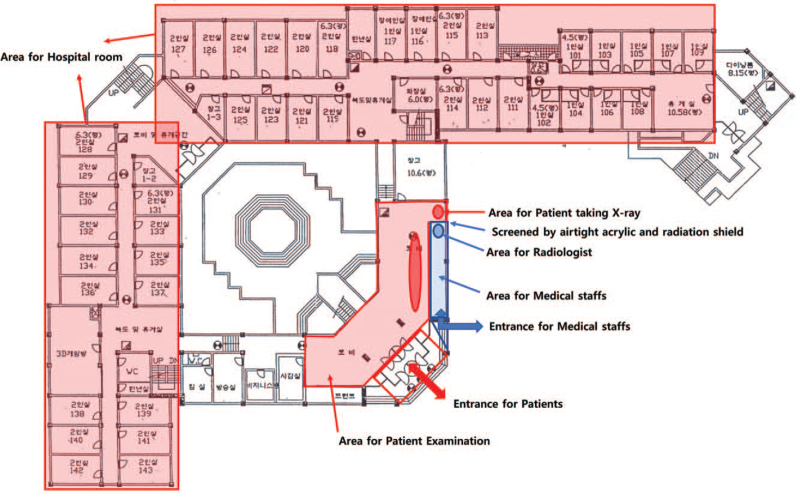
Building B for patient isolation 1st floor drawings.

**Figure 3:**
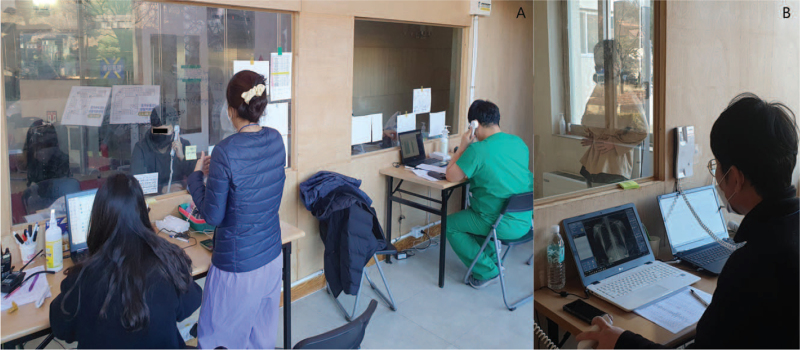
Noncontact medical room for identifying inpatient patients and performing x-ray, (A) Nurses and a doctor perform to consult and fill out medical card, (B) radiographer takes the chest x-ray.

A training institute for small-to-medium-sized businesses in Ansan was converted into a CTC. Building A is used by medical and administrative staff, and building B is used for patient isolation and examination, including X-rays. Building B has a total of 4 floors; there are 42 rooms on the first floor and 54 on the second, third, and fourth floors, with a total of 204 rooms. There are 36 single rooms and 168 double rooms, all of which are occupied by a single patient. However, as an exception, infants and parents entering the CTC together are allowed to stay in the same room. The medical staff and patients are separated on the first floor by bulkheads and acrylic windows to minimize the time and effort required by medical staff to put on and take off personal protective equipment (PPE) (Fig. 2). For the same reason, a bulkhead and acrylic window are installed in the X-ray room to separate patients and radiographers (Fig. 3).

### 2.3 Medical staff

In total, 13 nurses (1 head nurse, 1 charge nurse, and 11 regular nurses) work in the Ansan CTC. The head nurse and charge nurse perform regular daytime duties, while the 11 regular nurses have a schedule of 4 days of 1 day of daytime duty (8 am to 8 pm), 1 day of nighttime duty (8 pm to 8 am), and 1 day off. Each regular nurse takes responsibility for up to 6 patients. One senior medical specialist and 4 public health doctors work in the Ansan CTC. In total, 12 emergency-medicine specialists from Soonchunhyang University Bucheon Hospital work shifts at the Ansan CTC. Four public health doctors are rotated every 2 weeks. In preparation for emergencies, the senior specialist delivers training to nurses and public health doctors in basic life support and the use of defibrillators. In addition, 1 radiographer performs chest X-rays in the CTC.

### 2.4 Equipment and medications

One X-ray device is installed, and a biphasic defibrillator and emergency cart are available for emergencies. The emergency cart has drugs and appliances allowing the provision of advanced cardiovascular life support (Appendix 1, Supplemental Digital Content)). Patients are given a pulse oximeter, a tympanic membrane thermometer, and a sphygmomanometer, and instructions for their use are provided on the day of admission by a nurse wearing level D PPE. The drugs provided are mainly over-the-counter medications to treat symptoms (nonsteroid anti-inflammatory agents, acetaminophen, agents for gastro-intestinal symptom, agents for upper respiratory symptoms, and various ointments).

### 2.5 Operation of the CTC

Admissions take place once a day between 2 and 4 pm; if the number of patients for a given day is high, a morning admission session is added. The patients’ clothes are discarded on admission. During admission, patients are instructed to open their luggage as infrequently as possible. On admission, a nurse instructs each patient in the use of personal medical equipment (pulse oximeter, thermometer, and sphygmomanometer) and messenger applications (Kakaotalk, WhatsApp), and asks them to fill out an epidemiological survey form. A public health doctor then obtains the medical history (Fig. 3). Patients are discharged between 9 am and 10 am every day, with the precise time adjusted to prevent any overlap of the admission and discharge lines.

Morning and afternoon rounds (using video calls) are performed for all patients and those with special needs, respectively. A primary nurse checks the patient's self-recorded vital signs (blood pressure, heart rate, body temperature, and oxygen saturation), mood, level of discomfort, etc. When vital signs are unstable or symptoms have worsened, the doctor in charge decides whether to transfer the patient to the hospital. Video calls are conducted using patients’ smartphones; Google Translate or a foreign language call center are used to overcome language barriers. Most patients have a smartphone, but a public phone is available in the CTC in the event of problems using messenger applications.

**Figure 4:**
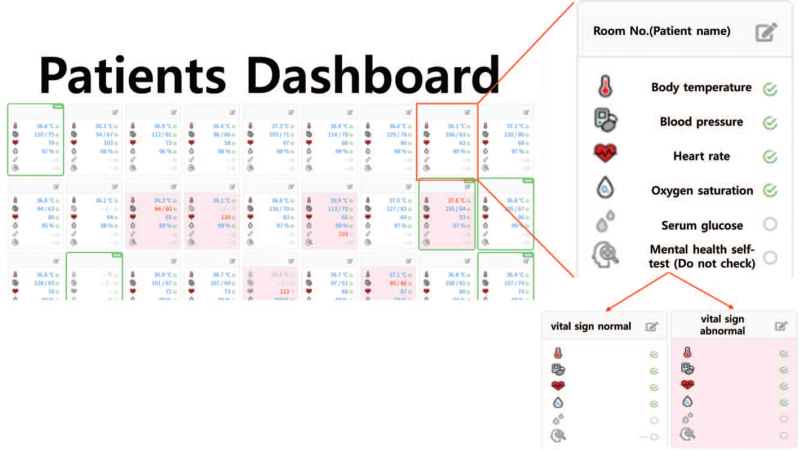
inPHR program screen, Doctors and nurses perform patient monitoring with inPHR program.

Patients self-reported twice a day using specialized mobile application (inPHR, SoftNet, Seoul, Korea) on vital signs (blood pressure, heart rate, body temperature, O2 saturation), chief complaints, and psychological conditions (anxiety, frustration, depression, and suicidal ideation) (Fig. 4).

Two or 3 chest X-rays are performed weekly, depending on the patient volume. Chest X-ray is typically performed once immediately after hospitalization, with follow-up imaging conducted in cases with abnormal findings or worsening symptoms. The PCR test is not routinely performed, but is available for patients willing to pay for it.

Patients select halal, vegetarian, or Korean meal options on admission. Military personnel wearing level D PPE serve meals 3 times a day. Medication is provided at meal times; if required at any other time, a nurse wearing level D PPE leaves the medication in front of the room.

All waste (including food waste) from patients is disposed of as medical waste, that is, is sterilized, disinfected or incinerated. Disinfection of the public spaces (hallways, elevator, and first-floor area) is performed 3 times per day. Disinfection of patient areas is performed after first ventilating the area, and takes place over the course of at least 1 hour for up to 24 hours after patient discharge. After cleaning, additional ventilation is performed for 6 to 24 hours before the arrival of new patients.

Patients are divided into groups A–C; in group A, all costs associated with COVID-19 quarantine are covered, while in group B lodging expenses are covered, but living and medical expenses are not. Group C patients cover the entire cost of the CTC stay themselves (Supplement Digital Content (Appendix 2, Supplemental Digital Content)).

## 3 Results

**Figure 5:**
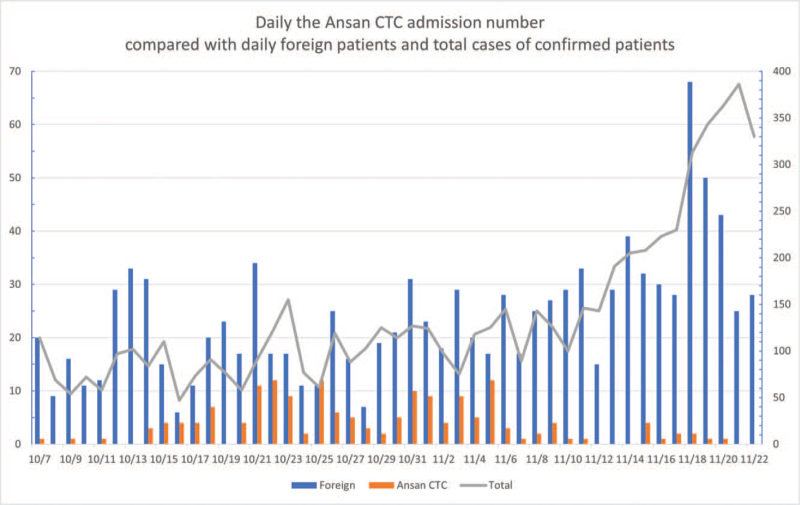
Time-line with daily the Ansan CTC admission number compared with daily foreign patients and total cases of confirmed patients. CTC = community treatment center.

In total, 315 patients were admitted to the Ansan CTC between 7 October and 22 November 2020; 145 of them were discharged from the CTC and 26 were transferred to other hospitals. During this period, the number of total cases of confirmed patients gradually increased toward the end of November, but the number of foreign patients and the number of admissions at the Ansan CTC were similar (Fig. 5).

In total, 60 (19.0%) patients were admitted directly to the CTC from an airport or quarantine facility, while 52 (16.5%) were admitted from a local health center, and 127 (40.4%) were admitted from residential facilities. For 76 (24.1%) patients, the hospitalization route was unknown. Among the 315 patients, 145 (84.8%) were discharged and 26 (15.2%) were transferred to other hospitals. In total, 31 (18.5%) patients received medication, 11 were admitted with fever and myalgia, 12 were admitted with COVID-19 symptoms other than fever, 10 were admitted due to comorbidities (hypertension, hepatitis B, or hyperthyroidism), and 3 were admitted for dysmenorrhea (Table 1).

**Table 1:** Patient characteristics.

Characteristics	Total, no. (%)
Total	168
Sex	
Female	42 (25.0)
Male	126 (75.0)
Age, yr	31 (25.5–39.0)
Length of stay, d	10.0 (10.0–11.0)
Route of admission	
Airport	40 (23.8)
Local health center	21 (12.5)
Residence facility	74 (44.0)
Missing value	33 (19.6)
Symptom at admission	
Fever	12 (7.1)
Cough	12 (7.1)
Sputum	6 (3.6)
Rhinorrhea	10 (6.0)
Sore throat	10 (6.0)
Myalgia	3 (1.8)
Anosmia	6 (3.6)
GI symptom	3 (1.8)
Initial CXR	
Pneumonia	4 (2.4)
Pleural effusion	2 (1.2)
Atelectasis	5 (3.0)
Other lesions	4 (2.4)
Normal	153 (91.1)
Medication	31 (18.5)
Outcome	
Discharge	145 (84.8)
Transfer	26 (15.2)
Reasons for transfer	Total N = 26
Uncontrolled fever (%)	4 (15.4)
Uncontrolled blood pressure (%)	12 (46.2)
Chest tightness (%)	3 (11.5)
Abnormal CXR (%)	4 (15.4)
Etc. (%)	3 (11.5)

CXR = chest x-ray, No. = number.

Next, 26 of 168 patients were transferred to other hospitals, mainly due to uncontrolled or worsening symptoms. There were 12 patients with uncontrolled blood pressure (46.2%), 4 with uncontrolled fever (15.4%), 4 with abnormal chest X-rays (15.4%), 3 with chest tightness (11.5%), and 3 others (11.5%) (Table 1).

**Figure 6:**
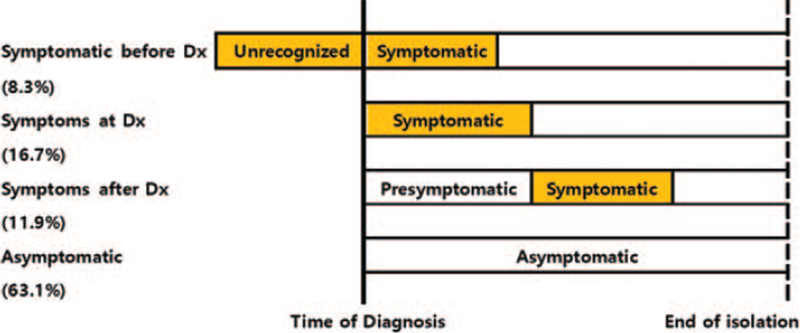
Time course in patients with COVID-19, patients with symptomatic before diagnosis is 8.3%, patients with symptoms at diagnosis is 16.7%, patients with symptoms after diagnosis is 11.9%, and patients with asymptomatic is 63.1%. COVID-19 = coronavirus disease-2019, Dx = diagnosis.

In total, 8.3% of patients had symptoms for a median of 3 days before COVID-19 diagnosis, while 16.7% had symptoms at the time of diagnosis and 11.9% had symptoms for a median of 1 day after diagnosis; 63.1% of patients were asymptomatic (Fig. 6).

## 4 Discussion

During the study period, among all overseas COVID-19 cases, 28.3% were admitted to the Ansan CTC; 84.8% of those patients were discharged and 15.2% were transferred to hospitals. The median length of stay was 10 days. Most transfers were decided within 1 to 2 days of CTC admission. In 18.5% of patients, medication was required to treat COVID-19 symptoms.

The Ansan CTC ensures efficient resource use by focusing on mild and asymptomatic COVID-19 patients, especially foreigners, in an environment combining elements of the hospital and home.

It can be disastrous if medical resource demand exceeds availability.^[14,15]^ Medical resources tend to be in particular demand during a pandemic.^[16,17]^ Victims of other types of disasters tend to be treated only once, whereas for pandemics at least 2 weeks of isolation and treatment may be needed.^[10]^ As a lack of medical resources increases casualties, effective preparation and deployment thereof is important.^[18,19]^ Recent reports have discussed pandemic situations in Korea and other countries in the context of surge capacity.^[5,10–12,20]^

During the COVID-19 pandemic, most countries have established self-quarantine procedures for the management of mild and asymptomatic patients.^[10,11]^ However, to prevent the spread of infection effectively, Korea has instead established CTCs in existing accommodation facilities to quarantine patients. Medical staff use video calls to monitor patients and provide the necessary treatment. Korea had achieved a low COVID-19 mortality rate of 2% by August 2020 due to the provision of both CTC and hospital care.^[21]^

Even during a pandemic, some people will continue to travel between countries.^[22]^ Managing the constant stream of COVID-19-infected individuals from other countries remains a challenge. Few studies have explored the management of foreign patients, but it has been demonstrated that mild and asymptomatic patients can be safely managed in CTCs.^[3,4,7,12]^ Thus, the Ansan CTC is designed exclusively for mild and asymptomatic foreign patients.

The Ansan CTC differs from other CTCs due to its use of non-face-to-face telemedicine to discuss all treatments with patients. Medical staff do not enter the patient zone, except in emergency situations; this reduces their workload. Second, routine PCR testing is not performed at the Ansan CTC due to the difficulties for medical staff of wearing level D PPE and the necessity of contacting patients for tests on an individual basis. At the Ansan CTC, patients are released from quarantine based on their clinical progress. To this end, chest X-ray is performed 2 or 3 times a week, depending on the volume of patients. For the Seoul CTC, which is used for South Korean patients and has a much higher workload than the Ansan CTC, PCR tests and chest X-rays are typically performed every 2 days.^[4]^ Third, the Ansan CTC provides patients with a variety of dietary options, including halal, vegetarian, and Korean. This is important because Muslims eat only halal food, Hindus cannot eat beef, and vegetarians obviously eat only vegetarian food.

Nearly 85% of patients are discharged from the Ansan CTC; around 15% are transferred to other hospitals, far higher than the rate of 2% to 10% reported by other studies.^[3,4,7,12]^ Compared to patients who are discharged, the patients transferred to other hospitals are older and have significantly higher systolic blood pressure. In more than half of the cases, patients are transferred either due to uncontrolled blood pressure or worsening COVID-19 symptoms. Along with stratifying by severity, comorbidities should also be considered. According to the KCDC criteria, patients with mild symptoms and 1 or more comorbidities are admitted to the Ansan CTC, but it is difficult to control high blood pressure with oral medications alone. Other reasons for transfer include mastitis, anxiety, and depressive episodes. There have been no reported deaths among patients discharged from CTCs, including the Ansan CTC.^[3,4,7,12]^ During the transfer process, it is difficult to determine bed availability because the medical staff at the Ansan CTC do not have access to real-time information on bed or hospital availability.

Based on the patient transfers at our CTC, pre-triage and resource availability are important considerations in the response to a disaster. Triage failure can lead to unnecessary transfer of patients, resulting in wasted medical resources. Through triage, CTCs can easily quarantine and manage mild and asymptomatic patients, thereby preventing overuse of hospital resources. CTCs use more resources compared to simply quarantining mild and asymptomatic symptoms in their own homes, as done in other countries. However, such self-quarantine may fail to prevent the transmission of infection^[23]^; CTCs better prevent COVID-19 transmission in South Korea compared to the measures used in other countries.^[2]^

Currently, the movement of citizens between countries is limited, but this should improve on resolution of the COVID-19 pandemic. However, this may increase the transmission of infections. This report could be instructive to that end, by demonstrating that a hybrid self-quarantine and hospitalization environment is an effective way to care for patients. It is important to provide suitable food for foreign COVID-19 patients in accordance with their country of origin, and to establish admission criteria and monitor their condition daily, including through video calls.

### 4.1 Limitations

The first limitation of this study is that the patients do not undergo blood tests, that is, there are no laboratory findings and the degree of viral load is unknown, so decisions regarding patient transfer are made based on symptoms alone. In addition, patients may give a false report of their symptoms and body temperature to ensure discharge. This could be addressed in the future by using Bluetooth devices to measure vital signs. Second, our CTC, located near Incheon International Airport, is designed for foreign patients, so the results may not be generalizable to other CTCs. Third, unlike some other CTCs, our center does not provide psychiatric counseling or medication due to difficulties with the storage of psychotropic drugs and communication in foreign languages. Patients with psychiatric issues are transferred to another hospital. Finally, to determine the need for surge capacity, further studies are required.

## 5 Conclusions

Even during the COVID-19 pandemic, individuals may have to travel between countries for various reasons, so a continuous influx of foreign patients with COVID-19 into South Korea is likely. CTCs can be an effective option for providing foreign patients with medical resources. This report should be instructive with respect to the operation of CTCs for foreign patients with COVID-19.

## Author contributions

**Conceptualization:** Hyun Noh.

**Data curation:** Han Bit Kim, Sangsoo Han, Heejun Shin, Young Hwan Lee, Kyung Min Lee, Jae Ryoung Kwak, Young Soon Cho, Hojung Kim, Hoon Lim, Gi Woon Kim, Eunkyung Eo, Hyun Noh.

**Formal analysis:** Han Bit Kim.

**Investigation:** Han Bit Kim.

**Supervision:** Hyun Noh.

**Writing – original draft:** Han Bit Kim.

**Writing – review & editing:** Han Bit Kim, Hyun Noh.

## Supplementary Material

Supplemental Digital Content

## Supplementary Material

Supplemental Digital Content
