# Supplementary figures and images for: Operation and management of a community treatment center using telemedicine for foreign patients with mild COVID-19 symptoms
Source: Medicine (Baltimore). 2021 Nov 24;100(47):e27948. doi: 10.1097/MD.0000000000027948 (PMC8615332; doi:10.1097/MD.0000000000027948)

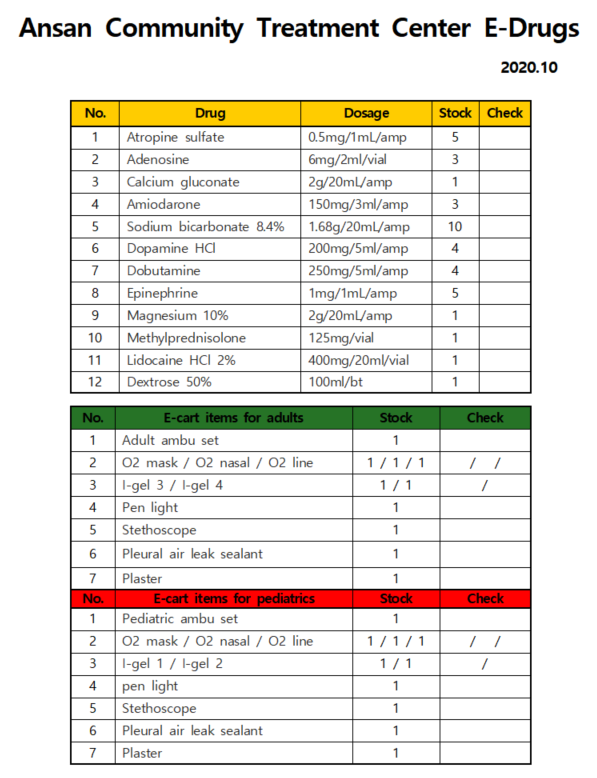

Supplement: Supplemental Digital Content [file medi-100-e27948-s001.tif]
