# Supplementary material for: Operation and management of a community treatment center using telemedicine for foreign patients with mild COVID-19 symptoms
Source: Medicine (Baltimore). 2021 Nov 24;100(47):e27948. doi: 10.1097/MD.0000000000027948 (PMC8615332; doi:10.1097/MD.0000000000027948)
Supplement: Supplemental Digital Content [file medi-100-e27948-s002.docx]

**Appendix 2. Classification for cost support by countries (11/01~11/30)**

| **Group A: All expenses arising from the treatment of COVID-19 isolation care** |
| --- |
| Gabon, Gambia, Greece, Guinea, Guinea, Guinea, Nigeria, Norway, New Zealand, Nicaragua, Taiwan, East Timor, Liberia, Russia, Lebanon, Romania, Libya, Lithuania, Malaysia, Mali, Morocco, Mozambique, Montenegro, Mongolia, Myanmar, Venezuela, Benin, Brunei, Saudi Arabia, Cyprus, Saint-Tome-Principe, Senegal, Serbia, Sudan, Slovakia, Iceland, Ireland, Armenia, Albania, Algeria, Estonia, Ethiopia, El Salvador, Britain, Yemen, Uganda, Uzbekistan, Italy, India, Japan, Equatorial Guinea, Central African Republic, Chad, Cameroon, Cabo Verde, Kazakhstan, Qatar, Kyrgyzstan, Canada, Kuwait, Trinidad and Tobago, Panama, Paraguay, Portugal, Poland, Australia |
| **Group B: Expenses only for isolation rooms, not for food and other medical expenses** |
| Guyana, Guatemala, Niger, Denmark, Germany, Latvia, Luxembourg, Macau, Mexico, Mauritius Moldova, Maldives, Democratic Congo, Bahrain, Bangladesh, Belgium, Belarus, Burundi, Burkina Faso, Northern Macedonia, Bosnia-Herzegovina, Bulgaria, Brazil, Sri Lanka, Spain, Spain, Slovenia, Syria, Singapore, Argentina, Azerbaijan, Afghanistan, Andorra, Angola, Ecuador, Oman, Austria, Jordan, Ukraine, Iran, Indonesia, Jamaica, China, Czech Republic, Chile, Costa Rica, Ivory Coast, Croatia, Tajikistan, Turkey, Pakistan, Peru, France, Finland, Philippines, Hong Kong |
| **Group C: All expenses on patient’s account** |
| Ghana, Namibia, South Sudan, South Africa, Netherland, Nepal, Dominica, Laos, Rwanda, Liechtenstein, Madagascar, Malawi, Mauritania, Malta, the United States, Bahamas, Vietnam, Belize and Bolivia, St. Kitts and Nevis, Suriname, Switzerland, Sierra Leone, the United Arab Emirates, Haiti, Antigua and Barbuda, Honduras, Uruguay, Iraq, Israel, Egypt, Zambia, Georgia, Zimbabwe, Cambodia, Kenya, Comoros, Colombia, Tanzania, Thailand, Togo, Tunisia, Papua New Guinea, the Republic of Fiji, Hungary, Vanuatu, Solomon Islands, Turkmenistan |
